# Hydrolysis of polydioxanone sutures in contact with hyaluronic acid of different concentrations: an in vitro study

**DOI:** 10.1590/0103-644020266626

**Published:** 2026-03-23

**Authors:** Juliano Bacon Modesto Setti, Ana Paula Christakis Costa, Flávia Pires Bretas Delaroli, Patrícia Moreira de Freitas, Victor Elias Arana-Chavez, Maristela Maia Lobo

**Affiliations:** 1 Master´s Degree in Odontology - Orofacial Harmonization. Postgraduation Program in Odontology. São Leopoldo Mandic College, Campinas, São Paulo, Brazil; 2 Federal University of Technology - Paraná (UTFPR), Graduate School of Electrical Engineering and Applied Computer Sciences (CPGEI), Curitiba, Paraná, Brazil; 3 Associate Professor. Department of Restorative Dentistry. Co-Chairman of the Special Laboratory of Lasers in Dentistry. School of Dentistry - University of São Paulo. São Paulo, São Paulo, Brazil; 4 Department of Biomaterials and Oral Biology. Postgraduate Program in Dentistry/Oral Biology. School of Dentistry - University of São Paulo. São Paulo, São Paulo, Brazil; 5 Associate Professor. Department of Postgraduation Program in Odontology. São Leopoldo Mandic College, Campinas, São Paulo, Brazil

**Keywords:** Polydioxanone, Hyaluronic Acid, Hydrolysis

## Abstract

Facial tissue repositioning with polydioxanone (PDO) thread is an effective alternative for stimulating collagen, treating sagging skin, and defining facial contours. When combined with hyaluronic acid (HA), further research is needed, as it has not been widely explored in the literature. This in vitro study investigated the hydrolysis pattern of spiculated PDO threads (I-Thread, 19G x 10 mm, USP-0) in contact with six different concentrations of HA (5, 12, 15, 17.5, 20, and 25 mg/mL). Ten cannulated PDO spiculated threads were fragmented into three and submerged in 1.0 mL of HA in test tubes at 37 °C, forming six groups: G1 (5 mg/mL NCTF 135 HA), G2 (12 mg/mL Volite), G3 (15 mg/mL Volbella), G4 (17.5 mg/mL Volift), G5 (20 mg/mL Voluma), and G6 (25 mg/mL Vollux). Optical and scanning electron microscopy and spectrophotometric degradation were performed at baseline, 1, 15, and 30 days. Statistical tests were applied at the 5% significance level. The results showed that the hydrolysis pattern of the threads varied with HA concentration. G1 (5 mg/mL NCTF 135 HA) maintained stability with lower absorbance values throughout the evaluation period (p < 0.013), suggesting less degradation. However, G6 (25 mg/mL Vollux) showed significantly higher absorbance and more pronounced structural changes (p < 0.001), indicating greater degradation. These findings suggest that HA concentration may influence the hydrolysis of PDO threads.

## Resumo

O reposicionamento de tecidos faciais com fio de polidioxanona (PDO) é uma alternativa eficaz no estímulo de colágeno, tratamento da flacidez e definição do contorno da face. Quando associado ao ácido hialurônico (HA) precisa de investigação, visto que não é amplamente explorado pela literatura. Este estudo in vitro investigou o padrão de hidrólise de fios espiculados PDO (I-Thread, 19G x 10 mm, USP-0) em contato com seis diferentes concentrações de HA (5, 12, 15, 17,5, 20 e 25 mg/mL). Dez fios espiculados de PDO canulados foram fragmentados em três e submersos em 1,0 mL de HA em tubos de ensaio a 37 °C, formando seis grupos: G1 (5 mg/mL NCTF 135 HA), G2 (12 mg/mL Volite), G3 (15 mg/mL Volbella), G4 (17,5 mg/mL Volift), G5 (20 mg/mL Voluma) e G6 (25 mg/mL Vollux). A microscopia óptica e eletrônica de varredura e espectrofotométrica da degradação foram realizadas no baseline, 1, 15 e 30 dias. Testes estatísticos foram aplicados considerando um nível de significância de 5%. Os resultados demonstraram que o padrão de hidrólise dos fios variou conforme a concentração de AH. O G1 (5 mg/mL de NCTF 135 AH) manteve-se estável com valores de absorvância mais baixos ao longo do tempo avaliativo (p < 0,013), sugerindo menor degradação. Em contrapartida, o G6 (25 mg/mL de Vollux) apresentou absorvância significativamente mais elevada e alterações estruturais mais pronunciadas (p < 0,001), indicando maior degradação. Esses achados sugerem que a concentração de HA pode influenciar a hidrólise dos fios de PDO.

**Figure:**
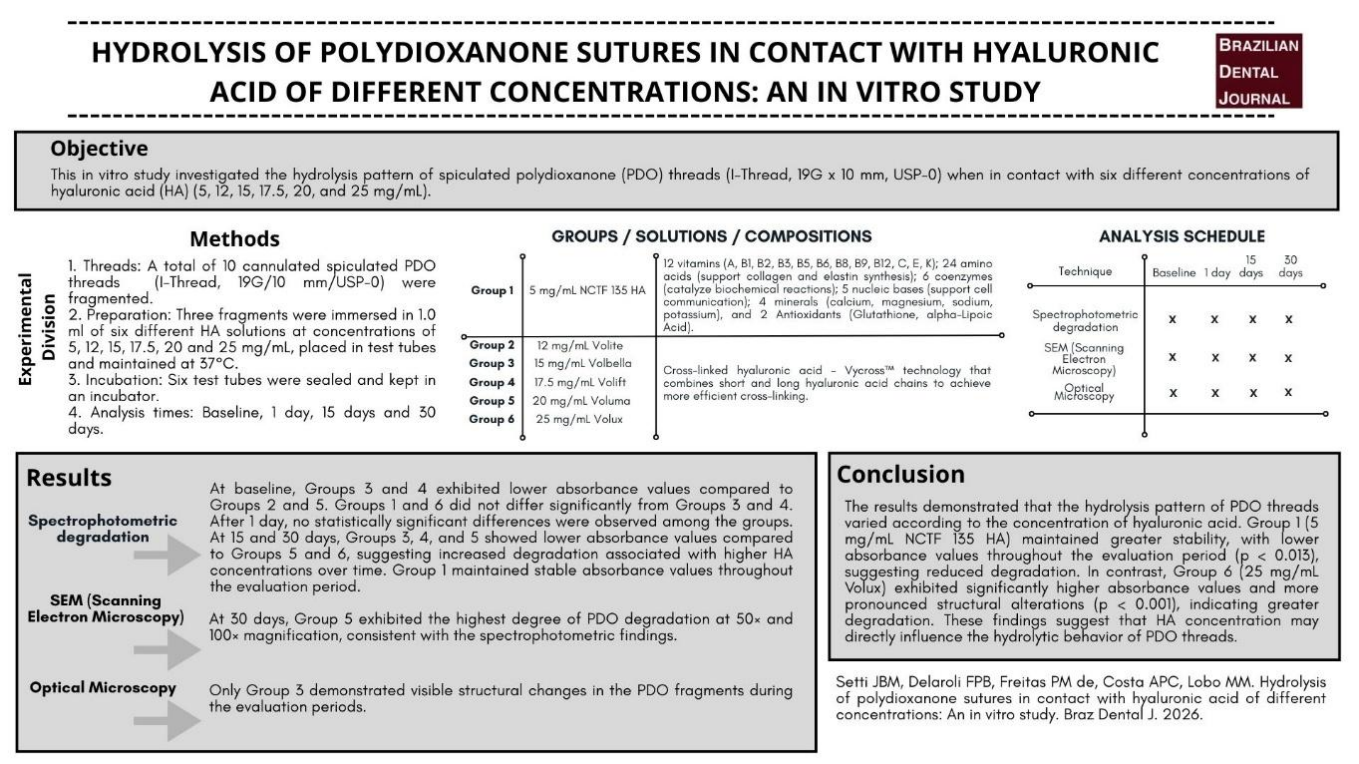

## Introduction

The aging process is a natural and inevitable occurrence that affects various aspects of a person's life, including psychological well-being. Everyone reacts uniquely to aging, and with increasing life expectancy, minimally invasive aesthetic products and techniques have emerged to delay the appearance of aging and promote a more youthful facial appearance ^1^ ^,^ ^2^.

Polydioxanone (PDO) sutures have been used safely and routinely in surgical procedures for over three decades. These sutures consist of highly flexible, absorbable synthetic monofilament polymers made from para-dioxanone monomers, which degrade in the body through non-enzymatic hydrolysis. They come in different shapes and sizes and are indicated for minimally invasive facelifts of the upper, middle, and lower thirds of the face and neck ^3^. 

With a considerable ability to maintain tension for a prolonged period compared to other support sutures, PDO sutures remain in the tissue for the time needed to induce local scarring and the formation of new collagen ^1^ ^,^ ^4^. Once inserted into the skin, the sutures maintain a support structure, allowing the facial tissues to be repositioned. This structural lifting and firming of the skin is known as the lifting effect and is complemented by the stimulation of collagen and elastin production, which improves the appearance and texture of the skin and smooths the facial contour ^1^ ^,^ ^2^.

This approach allows the sutures to deliver short-term results ^5^, providing efficacy in dermoscopy, filling, and rejuvenation, results often not achieved by other techniques ^2^. Furthermore, a remarkable aspect of PDO sutures is their ability to maintain tension until the scar volume is firmly established. Aesthetic results are maintained for an appropriate period, during which these sutures can retain up to 70% of the tension for up to 28 days after treatment and are completely absorbed within a range of 128 to 238 days after initial insertion ^1^. 

Following the successful use of PDO sutures for facial collagen biostimulation, new therapeutic approaches have developed protocols using hyaluronic acid (HA), thereby improving facial support, increasing self-esteem, and enhancing the patient's quality of life. For example, in the context of skin aging, related to chronological age and a decrease in dermal collagen, the combination of active ingredients with PDO sutures has emerged as a viable alternative for facial rejuvenation, resulting in stimulation of collagen synthesis, formation of thick and organized fibers, restoration of dermis quality, reducing hyperpigmentation, and promoting neocollagenesis ^2^. 

In the study of the management of aging, there is still limited scientific evidence to support the beneficial association between PDO sutures and HA-based fillers in different crosslinks, as well as the impact on the hydrolysis of these sutures. In this context, the literature lacks experimental studies that systematically evaluate how different concentrations and crosslinking levels of HA influence the hydrolysis process and the structural integrity of the threads. This lack of data compromises the establishment of safe, evidence-based protocols. This in vitro study investigated the hydrolysis pattern of spiculated PDO threads (I-Thread, 19G x 10 mm, USP-0) in contact with six different concentrations of HA (5,12,15,17,5,20, and 25 mg/mL).

### Polydioxanone (PDO) threads

The first procedures using facial lifting threads were introduced by Sulamanidze in 1998, with Aptos threads, inaugurating a minimally invasive approach to the rejuvenation of soft tissues in the face ^6^ ^,^ ^7^. Based on this innovation, several techniques and materials were developed, such as Woffles sutures, Waptos sutures, Isse unidirectional sutures, and Silhouette sutures. However, the use of permanent sutures caused concern about delayed complications, motivating the transition to absorbable threads capable of producing a temporary lifting effect with greater safety. In this context, polydioxanone (PDO) threads have become the most widely used in aesthetic procedures ^8^ ^,^ ^9^ ^,^ ^10^ ^,^ ^11^ ^,^ ^12^.

Introduced in 1982, PDO is a monofilament synthetic polymer (C₄H₆O₃) widely used in medical practice due to its biocompatibility, low toxicity, and chemical stability ^13^. It is an absorbable material whose degradation occurs by hydrolysis, producing biocompatible by-products that are subsequently metabolized into carbon dioxide and water, providing predictability and safety for clinical use, since this process is completed coincidentally with the tissue healing period ^1^ ^,^ ^13^ ^,^ ^14^.

The physical and mechanical properties of PDO, such as high flexibility and tensile strength, enable its application in areas subject to dynamic mobility, such as the face, maintaining adequate support until healing is consolidated ^15^. When provided with spicules, the threads take on an additional role as biostimulators, inducing neocollagenesis and providing results that can last from 18 to 24 months, even after their complete absorption ^16^. Complication rates are generally low, although events such as pain, ecchymoses, asymmetry, skin irregularities, infection, or nodule formation can occur ^7^ ^,^ ^17^.

The scientific literature offers consistent support for the effectiveness of PDO in aesthetic applications. Comparative studies have shown significant differences between commercial brands in terms of mechanical resistance and degradation profile ^18^, and histological research demonstrates that thread insertion promotes beneficial tissue remodeling, including neocollagenesis, superficial fat reorganization, tissue contraction, and improved vascularization ^19^. In addition, clinical analyses demonstrate high patient satisfaction and long-lasting results in facial folds and areas of sagging ^7^ ^,^ ^20^.

### Hyaluronic acid (HA) and its association with PDO threads

Hyaluronic acid (HA) is a dermal filler widely used in contemporary aesthetic practice due to its versatility, safety, and ability to modulate tissue volume in a predictable manner ^21^. Manufacturers offer different formulations, which vary in terms of HA concentration, degree of crosslinking, particle size, cohesiveness, and extrusion force, parameters that directly influence clinical performance ^5^.

Naturally present in the body, HA is composed of repetitive units of D-glucuronic acid and N-acetyl-D-glucosamine, possessing high hydrophilicity capable of retaining up to a thousand times its weight in water, a fundamental property for tissue turgor and hydration ^22^ ^,^ ^23^. In the skin, it is abundantly synthesized by fibroblasts and keratinocytes, integrating the extracellular matrix and contributing to dermal viscoelasticity ^24^.

As a dermal filler, in its crosslinked form, it has greater resistance to enzymatic degradation, which corrects furrows, restores volume, reshapes contours, and treats scars, with a clinical duration of between six and eighteen months ^5^ ^,^ ^25^. Crosslinking technology, often based on BDDE, is crucial for its stability, elasticity (G'), and cohesiveness, modulating its indication for superficial or deep planes ^26^ ^,^ ^27^. Studies also show that HA can stimulate fibroblasts and modulate collagen production through receptors such as CD44 and RHAMM, contributing to tissue quality ^28^.

Given the demands related to skin aging, the combination of techniques that stimulate collagenesis has become a relevant strategy in aesthetic practice, with the association between HA and PDO standing out. In this integrated approach, PDO threads promote tissue traction and remodeling, while HA corrects volumetric deficiencies and optimizes skin hydration, favoring the treatment of sagging, deep furrows, and facial contour disharmonies. However, authors emphasize the need for standardized protocols and controlled studies that evaluate the longevity, safety, and biochemical interaction between these biomaterials ^29^ ^,^ ^30^.

Although widely used in clinical practice, the association between HA and PDO has been the subject of experimental investigations that point to possible unfavorable interactions. Suárez-Vega et al. (2019) ^29^ demonstrated that non-crosslinked HA can accelerate the hydrolytic degradation of polydioxanone, with noticeable structural changes already in the first 24 hours, including increased interlamellar spaces, loss of pigmentation, fibril rupture, and disorganization of the polymer architecture. After 72 hours, it was observed that the greater hydrophilicity of HA acted as a catalyst for PDO hydrolysis. Subsequently, the same group described a clinical case in which the inadvertent combination of the two products resulted in accelerated degradation of poorly positioned PDO threads, avoiding the need for surgical removal ^31^. In addition, Zhou et al. (2023) ^32^ demonstrated that PDO microspheres have high biocompatibility, rapid biodegradation, and robust collagen stimulation, comparable or even superior to that of HA, reinforcing the potential of PDO as a promising biomaterial beyond its applications in traction threads.

In contrast, recent clinical studies highlight the relevant benefits of the combination of HA and PDO when applied in a planned manner. Wan et al. (2024) ^33^ observed significant volumetric restoration and improvement in the contour of the middle third, with a measurable reduction in facial width over 24 months. In addition, the authors reported high patient satisfaction, with 9 out of 11 individuals reporting continuous improvement. Altmayer & Szomolai (2025) ^34^ also attribute the prolonged effects to neocollagenesis induced by PDO threads, which enhances and sustains the results provided by HA.

From a mechanical perspective, Marques (2024) ^35^ describes that HA can accelerate the biodegradation of PDO threads, contributing to faster tissue remodeling. Its hydrophilic characteristic also improves skin hydration and elasticity, a finding corroborated by Silva et al. (2023) ^30^. Regarding safety, Wan et al. (2024) ^33^ and Park et al. (2024) ^36^ report a favorable profile, with a low incidence of adverse events and advantages inherent to minimally invasive procedures, such as shorter recovery time and lower risk of complications.

Despite the reported benefits, the possibility of accelerated degradation of the threads in the presence of HA-especially when both materials come into direct contact on the same tissue plane- can compromise the durability of the results ^35^. Thus, the findings converge on the importance of thoroughly understanding the interactions between biomaterials before proposing therapeutic combinations, reinforcing that, although the combined use of HA and PDO may be advantageous in specific clinical situations, its application requires careful planning and individualization of approaches.

## Materials and Methods

This in vitro study selected 10 cannulated PDO spiculated sutures (I-Thread, 19G x 10mm, USP-0) and six types of HA at concentrations of 5, 12, 15, 17.5, 20, and 25 mg/mL. The sample size was estimated by repeated measures ANOVA, considering a statistical power (1-β) of 80% and a significance level (α) of 5% (0.05). 

**Figure 1:**
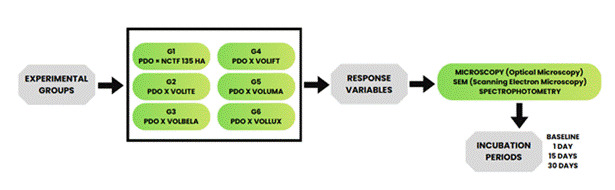
Flowchart of the study.

**Figure 2:**
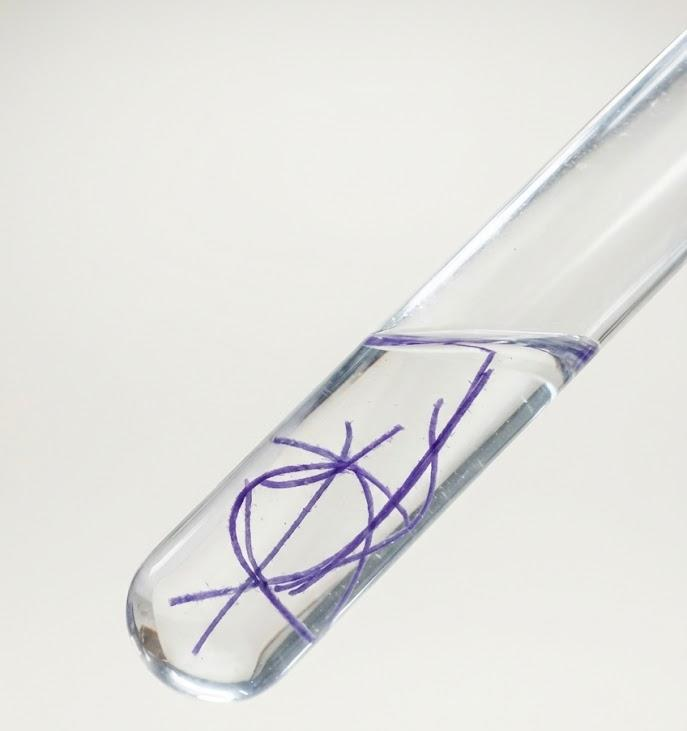
PDO threads are immersed in AH.

Each PDO suture was fragmented into three parts and submerged in 1.0 mL of HA at different concentrations. The test tubes were then placed in a study maintained at 37°C and incubated for Baseline, 1, 15, and 30 days. A total of six groups were formed: G1 with PDO and HA without crosslinking at a concentration of 5 mg/mL NCTF 135 HA (Filorga); G2 with PDO and HA crosslinked at a concentration of 12 mg/mL Volite (Allergan); G3 with PDO and HA crosslinked at a concentration of 15 mg/mL Volbella (Allergan); G4 with PDO and crosslinked HA at a concentration of 17.5 mg/mL Volift (Allergan); G5 with PDO and crosslinked HA at a concentration of 20 mg/mL Volume (Allergan); and G6 with PDO and crosslinked HA at a concentration of 25 mg/mL Vollux (Allergan). A control group with immersion in a neutral solution (PBS or distilled water) was not included. It was decided to exclusively compare different concentrations of HA, as the aim was to specifically evaluate the direct impact of these products on the hydrolysis of PDO (Figures 1 and 2).

NCTF 135 HA (Filorga) is a poly-revitalizing solution that includes 55 active ingredients and non-cross-linked hyaluronic acid at a concentration of 5mg/ml. The components include: 12 vitamins, which stimulate vital cell functions; 24 amino acids, which promote the synthesis of proteins such as collagen and elastin; 6 coenzymes, which catalyze biochemical reactions in tissue; 5 nucleic bases, which activate cell communication; 4 minerals, which compensate for skin deficiencies; 2 antioxidants, which reduce the synthesis of free radicals. The other HAs used a technology known as Vycross^TM,^ which combines short and long chains of hyaluronic acid for better crosslinking (Table 1).

**Figure 3:**
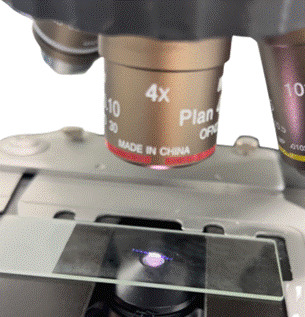
Evaluation under an optical microscope.

At baseline, 1, 15, and 30 days, each fragment of PDO suture from each group was removed from the test tube, placed in a petri dish, and prepared for subjective and structural analysis by optical microscopy, using 4x, 10x, and 20x magnification parameters (Figure 3). After this analysis, the sutures were returned to the test tubes. Next, specimens were mounted on aluminum stubs coated with gold sputtering (Balzers SDC-050) and examined using a scanning electron microscope (SEM, Leo 430), operating at 15 kV, at baseline and 30 days. Furthermore, the liquids were analyzed by spectrophotometry in 96-well microplates (Biotek Epoch), which quantitatively measured, at 400 nm, the absorbance of light by the solutions at baseline, 1, 15, and 30 days. The light absorption was proportional to the amount of substance absorbed. All microscopic analyses were carried out by a single, previously calibrated observer.

These results were evaluated statistically. Median differences were compared using the Kruskal-Wallis test, time effects were compared using the Friedman test, and multiple comparisons were performed using the Student-Newman-Keuls test. For this, the SPSS 23 (SPSS INC., Chicago, IL, USA) and BioEstat 5.0 (Mamirauá Foundation, Belém, PA, Brazil) programs were used, considering a significance level of p-value ≤ 0.05.

**Table 1:** Chemical composition of the intradermal substances of the HA.

Substances	Types	Components
NCTF 135 HA (5 mg/mL - Filorga)	Non-cross-linked	12 vitamins (A, B1, B2, B3, B5, B6, B8, B9, B12, C, E, K); 24 amino acids (support collagen and elastin synthesis); 6 coenzymes (catalyze biochemical reactions); 5 nucleic bases (support cell communication); 4 minerals (calcium, magnesium, sodium, potassium), and 2 Antioxidants (Glutathione, alpha-Lipoic acid).
Volite (12 mg/mL)	Crosslinked hyaluronic acid - Vycross™ technology that combines short and long hyaluronic acid chains to achieve more efficient crosslinking.	12 mg/mL of crosslinked hyaluronic acid, 0.3% lidocaine and Physiological buffer solution.
Volbella (15 mg/mL)		15 mg/mL of crosslinked hyaluronic acid, 0.3% lidocaine and Physiological buffer.
Volift (17,5 mg/mL)		17.5 mg/mL of crosslinked hyaluronic acid, 0.3% lidocaine, and Physiological buffer.
Voluma (20 mg/mL)		20 mg/mL of crosslinked hyaluronic acid, 0.3% lidocaine and Physiological buffer.
Volux (25 mg/mL) (Allergan)		25 mg/mL of crosslinked hyaluronic acid, 0.3% lidocaine and Physiological buffer.

## Results

At baseline, in the groups whose PDO strands were immersed in HA at concentrations of 15 mg/mL (Volbella, G3) and 17.5 mg/mL (Volift, G4), the absorbance values were significantly lower than those found in the groups in which the sutures were in contact with concentrations of 12 mg/mL (Volite, G2) and 20 mg/mL (Voluma, G5) of HA (p=0.021). The PDO fragments at concentrations of 5 mg/mL (NCTF 135 HA, G1) (p<0.001) and 25 mg/mL (Volux, G6) (p<0.001) showed no difference between the groups with 15 mg/mL (Volbella, G3) (p=0.011) and 17.5 mg/mL (Volift, G4) (p=0.359). These last two groups also had no significant difference concerning the group whose suture was in contact with HA at a concentration of 20 mg/mL (Voluma, G5) (p=0.002) (Table 2).

When measuring after 1 day, there was no statistically significant difference between the absorbance values when comparing the six groups tested. However, after 15 days and 30 days, as can be seen in Table 1, the absorbance in the groups whose PDO sutures had contact with HA at concentrations of 12 mg/mL (Volite, G2), 15 mg/mL (Volbella, G3) and 17.5 mg/mL (Volift, G4) was significantly lower than in the groups that received the acid at concentrations of 20mg/mL (Voluma, G5) and 25 mg/mL (Volux, G6). However, the group whose sutures were in contact with HA at a concentration of 5mg/mL (NCTF 135 HA, G1) showed absorbance values that did not differ significantly from those found in any of the other five groups (Table 2).

All these findings can be found in Table 1, and the identifications by lower case letters (a, b, c) show the differences found between the absorbance values at the 4 evaluation times, considering each concentration of HA. Moreover, the capital letters (A, B, C) indicate the differences found between the absorbance values in the groups evaluated, considering each concentration of HA. 

**Figure 4:**
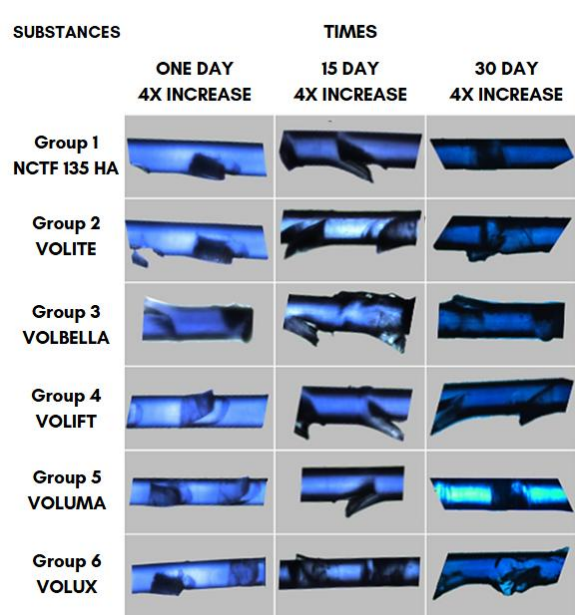
Optical microscopy images showing the structural aspects of the six products at the three analysis points.

From the analysis carried out under the optical microscope, when checking the structure of the PDO sutures, the one that showed a structural variation was the PDO sutures associated with HA at a concentration of 15 mg/mL (Volbella, G3). However, this finding may be indicative of an alteration due to HA, since in the absorbance analysis, at 15 days, the absorbance was 0.149 nm, or it may correspond to a structural failure of the suture, due to manufacturing or initial fragmentation for this experiment (Figure 4).

**Table 2:** Medians, minimum, and maximum absorbance values (nm) of groups in which PDO sutures were exposed to different concentrations of hyaluronic acid over time.

Groups	Baseline	Day 1	Day 15	Day 30
PDO suture + 5 mg/mL hyaluronic acid (NCTF 135HA) (G1)	0.064 (0.051; 2.970) ABa	0.165 (0.115; 3.092) Ab	0.326 (0.211; 3.222) ABCc	0.230 (0.198; 3.188) ABCbc
PDO suture + 12 mg/mL hyaluronic acid (Volite) (G2)	0.0470020 (0.036; 2.945) Cb	0.045 (0.038; 2.965) Aa	0.060 (0.051; 2.951) ABab	0.060 (0.051; 2.933) ABab
PDO suture + 15 mg/mL hyaluronic acid (*Volbella*) (G3)	2.002 (1.951; 2.337) Aa	0.053 (0.043; 2.975) Ab	0.149 (0.125; 2.994) Aa	0.137 (0.116; 2.992) Aa
PDO suture + 17.5 mg/mL hyaluronic acid (*Volift*) (G4)	0.052 (0.046; 2.966) Aa	0.066 (0.055; 2.978) Aa	0.535 (0.046; 2.925) Aa	0.535 (0.046; 2.911) Aa
PDO suture + 20 mg/mL hyaluronic acid (*Voluma*) (G5)	0.987 (0.924; 3.876) BCab	1.935 (1.710; 2.525 )Ab	0.666 (0.612; 3.457) BCa	0.671 (0.617; 3.504) BCab
PDO suture + 25 mg/mL hyaluronic acid (*Volux*) (G6)	0.157 (0.132; 3.029) ABa	0.206 (0.180; 3.104) Aab	1.140 (1.011; 3.654) Cb	1.015 (0.911; 3.574) Cb

For intergroup (difference between columns represented by a capital letter) comparisons at baseline - p = 0.021; at day 1 - p = 0.065; at day 15 - p = 0.013; at day 30 - p = 0.014. For intragroup (difference between rows represented by a lowercase letter) comparisons over time for each group: NCTF 135HA - p < 0.001; Volite - p = 0.002; Volbella - p = 0.011; Volift - p = 0.359; Voluma - p = 0.002; Volux - p < 0.001. Source: Author’s work.

**Figure 5:**
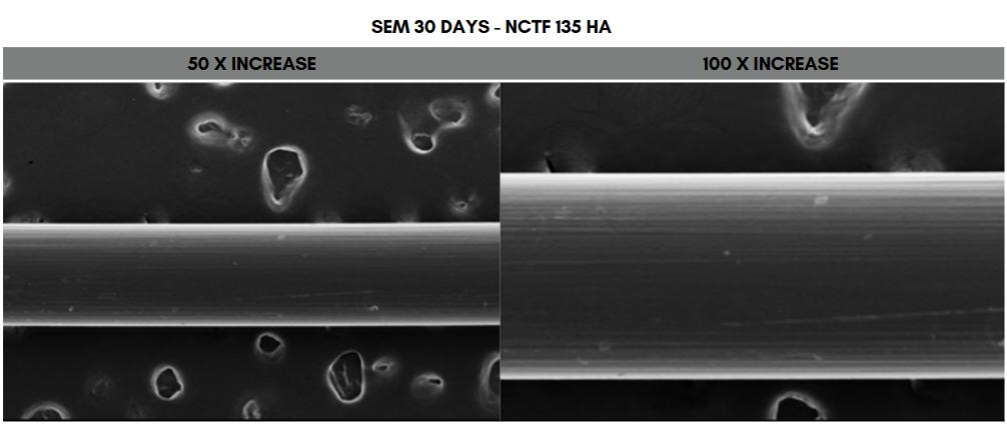
Scanning electron microscopy (SEM) images of PDO threads immersed in NCTF 135 for 30 days, with magnifications of 50x and 100x.

**Figure 6:**
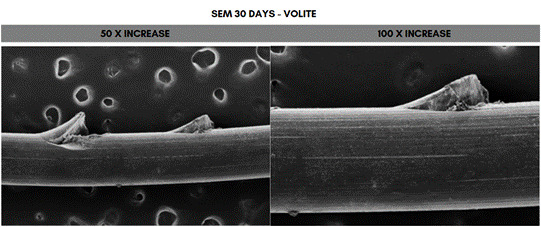
Scanning electron microscopy (SEM) images of PDO threads immersed in Volite for 30 days, with magnifications of 50x and 100x.

**Figure 7:**
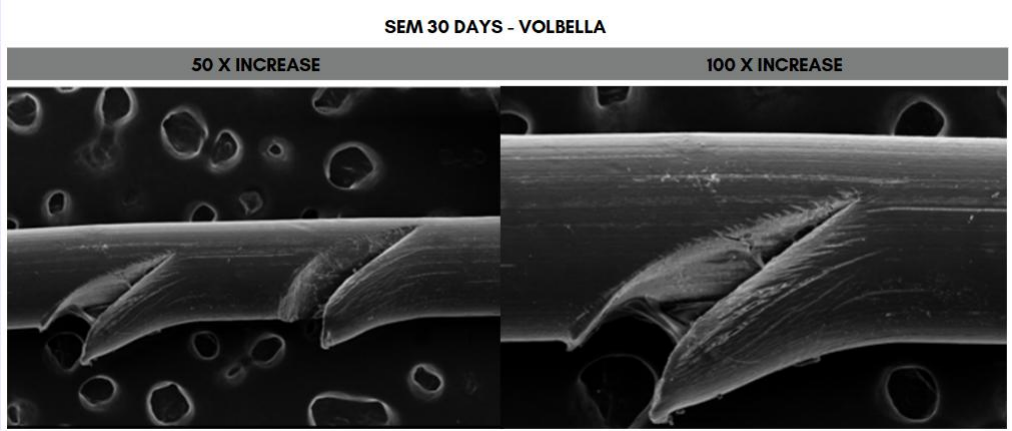
Scanning electron microscopy (SEM) images of PDO threads immersed in Volbella for 30 days, with magnifications of 50x and 100x.

**Figure 8:**
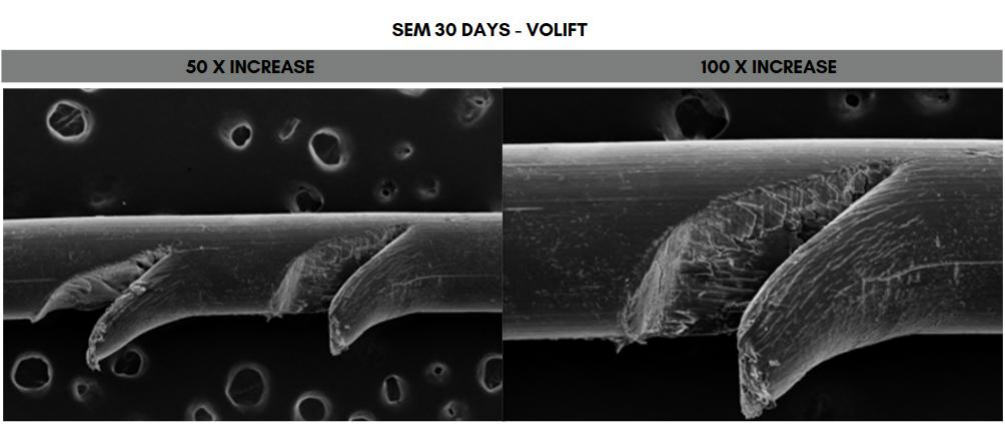
Scanning electron microscopy (SEM) images of PDO threads immersed in Volift for 30 days, with magnifications of 50x and 100x.

**Figure 9:**
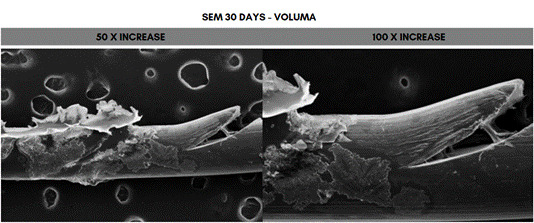
Scanning electron microscopy (SEM) images of PDO threads immersed in Voluma for 30 days, with magnifications of 50x and 100x.

**Figure 10:**
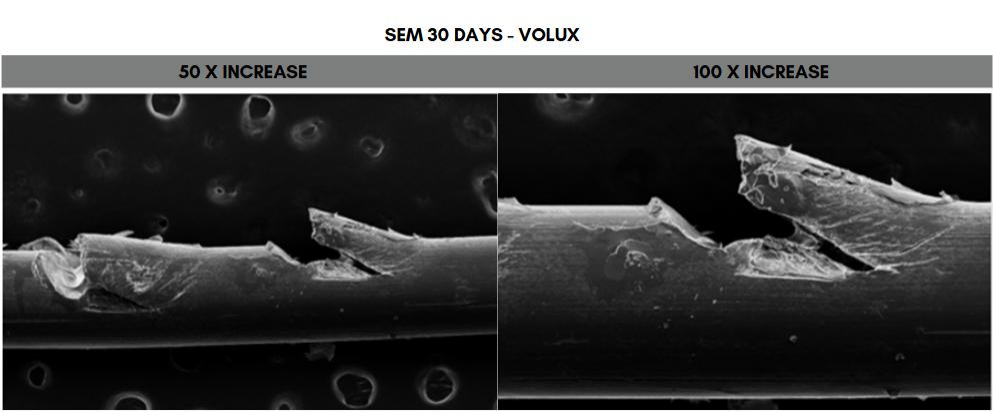
Scanning electron microscopy (SEM) images of PDO threads immersed in Volux for 30 days, with magnifications of 50x and 100x.

Scanning electron microscope (SEM) analysis of the PDO sutures associated with the different concentrations of HA at 50- and 100-times magnification, 30 days after immersion. The fragment that showed the greatest degradation was the one immersed in 20 mg/mL (Voluma, G5), while the one that showed the least degradation was the fragment immersed in 5 mg/mL (NCTF 135 HA, G1) (Figures 5, 6, 7, 8, 9, and 10). 

## Discussion

This in vitro investigation evaluated the hydrolysis of spiculated PDO threads in contact with different concentrations of HA, aiming to determine whether filler concentration influences the structural stability of PDO. The findings showed that HA concentrations can affect the hydrolysis of spiculated PDO threads, especially higher concentrations such as 20 mg/mL and 25 mg/mL, which induced greater degradation of the threads, while 5 mg/mL HA (NCTF 135 HA) showed greater stability over time. These results provide new and relevant evidence for clinical practice, since few experimental studies have directly evaluated the interaction between HA and PDO.

Given the results obtained in this investigation, the concentrations of HA used can influence the hydrolysis of PDO sutures. Therefore, the concentration of HA is an important factor to consider in aesthetic procedures involving the use of PDO threads ^2^. To clarify this discussion, it is necessary to contextualize the structural and functional properties of polydioxanone, widely recognized as a synthetic, biocompatible polymer susceptible to gradual hydrolytic degradation and sensitive to the presence of highly hydrated environments, as demonstrated by Sabino et al. (2000) ^37^ and reinforced by Zhou et al. (2023) ^32^.

In addition to the studies previously cited, recent evidence from biomaterials science reinforces that the hydrolysis of absorbable polymers, such as polydioxanone, is strongly influenced by highly hydrated environments and the presence of hydrophilic macromolecules. Middleton and Tipton (2000) ^38^ demonstrated that synthetic polymers used in medical applications exhibit degradation rates directly related to water diffusion into the polymer matrix, accelerating the cleavage of ester connections. This behavior was later confirmed by Vert et al. (2012) ^39^, who highlighted the importance of polymer-medium interaction in the structural stability of absorbable biomaterials.

Specific studies on absorbable sutures indicate that increased local moisture and water content accelerate the loss of mechanical strength and structural fragmentation. Chu et al. (2017) ^40^ observed that polydioxanone sutures subjected to highly hydrated media showed a significant reduction in tensile strength even before complete macroscopic degradation. Similar findings were described by Vieira et al. (2014) ^41^, who demonstrated that environments rich in hydrophilic polymers favour water penetration into the PDO matrix, intensifying the hydrolysis process.

In the context of hyaluronic acid, additional studies corroborate that its high affinity for water can modify the tissue and biomaterial microenvironment. Sundaram et al. (2015) ^42^ and Flynn et al. (2011) ^43^ highlighted that HA formulations with higher concentration and greater cohesiveness have greater water retention capacity, which can negatively impact adjacent materials sensitive to hydrolysis. More recently, Micheels and Eng (2018) ^44^ reinforced that the physicochemical interaction between HA and absorbable biomaterials should be considered when defining clinical application plans. Thus, the findings of the present study are consistently supported in contemporary literature, reinforcing that the concentration of hyaluronic acid is a critical factor in the stability of polydioxanone. These data expand the scientific basis that supports the need for careful planning in the association between HA and PDO threads, especially when there is a risk of direct contact between biomaterials.

Performing tests on the properties of PDO threads has become crucial both in the manufacturing stage and in clinical decisions regarding the choice of materials ^3^ ^,^ ^13^. An ideal thread should be biologically inert, resistant to traction, cause no harmful effects to the tissue, be easy to handle by the dental surgeon, not allow bacterial growth, have high tensile strength, be easy to sterilize, cause no carcinogenic action, and be absorbed after performing its function ^13^ ^,^ ^45^. Spiculated PDO threads have good mechanical properties and excellent anchoring capacity, which generates a force of action that opposes the traction movement of the tissue, making them suitable for minimally invasive facelift procedures ^15^. The tensile strength of this thread is consistent with the combination of the following factors: the diameter of the sutures, the manufacturing design, and the properties of the threads. However, different PDO sutures have different mechanical characteristics, such as molded sutures, which are more fragile than spiculated threads ^15^, presenting distinct profiles of resistance and degradation, reinforcing the need for studies such as this one. Given these characteristics, PDO sutures fill the gap between all other rejuvenation procedures ^46^.

PDO and HA threads are often used independently in aesthetic procedures, but their direct interaction has received little attention in the literature. Recent studies, such as Silva et al. (2023) ^30^, highlight the aesthetic benefits of combined use, but reinforce that the lack of structural studies on such interactions represents a scientific gap. In the present study, it was observed that HA, being highly hydrophilic, can physically interact with the polymeric structure of PDO, absorbing water and accelerating its degradation, a phenomenon consistent with Suárez-Vega et al. (2019) ^29^.

HA has a significant role in improving tissue hydration ^2^ ^,^ ^30^ ^,^ ^47^ and inflammatory modulation, acting on fibroblasts through receptors such as CD44 and RHAMM ^21^ ^,^ ^22^ ^,^ ^23^ ^,^ ^28^ ^,^ ^47^. Therefore, the interaction between HA and PDO will depend on both the chemical properties of HA and the structural conditions of the suture. It is important to note that studies such as Kablik et al. (2009) ^48^ and Fagien et al. (2019) ^27^ demonstrate that the concentration and rheological characteristics of HA influence its viscosity, cohesiveness, and water absorption capacity, which may explain the increase in hydrolysis observed here ^5^ ^,^ ^27^ ^,^ ^28^ ^,^ ^49^.

Another relevant point concerns the metabolism of HA. Being a hydrophilic polymer, its presence in high concentrations can intensify the entry of water into the PDO microfibrils, as observed in studies by Ping Ooi & Cameron (2002) ^50^. This is consistent with the structural observations of this study under the microscope, showing longitudinal cracks, spicula wear, and increased interfibrillar spaces at concentrations above 12 mg/mL. These findings also coincide with Suárez-Vega et al. (2019) ^29^, who demonstrated degradation within the first 24-72 hours of contact.

Comparison with contemporary clinical data reinforces the practical relevance of the findings. Wan et al. (2024) ^33^ demonstrated that PDO and HA can be used complementarily, with excellent aesthetic results and improved facial contour when applied in different planes, avoiding direct contact. Similarly, Altmayer & Szomolai (2025) ^34^ observed that PDO prolongs the clinical action of HA through neocollagenesis, as long as the biomaterial does not interact directly. In line with this, Marques (2024) ^35^ suggests that HA can accelerate the biodegradation of PDO when there is direct structural proximity, corroborating the behavior observed in the present study. 

In this study, it was observed that HA at 5 mg/mL (NCTF 135 HA) showed less degradation when compared to the other concentrations. This finding partially diverges from the hypothesis of Suárez-Vega et al. (2019) ^29^, who observed accelerated degradation in non-crosslinked HA. The difference can be attributed to the presence of vitamins, amino acids, and nucleotides in NCTF, which modifies osmotic behavior and interaction with the thread. Studies by Siqueira & Canevassi (2022) ^2^ agree that NCTF modulates hydration and fibroblast response differently than pure HA. 

The degradation observed, especially in threads immersed in 20 and 25 mg/mL HA, suggests that high concentrations increase the hydrolytic effect on PDO. In other words, water that penetrates the structure causes dissolution. This behavior is consistent with studies of absorbable biomaterials ^32^ ^,^ ^51^, which show that highly hydrated environments accelerate the breakdown of ester bonds. The present study further reinforces that PDO degradation does not occur linearly but depends on variables such as concentration, HA structure, and exposure time, requiring further investigation to analyze in vivo behavior-tissue hydration, HA turnover, and inflammatory response can modify this process.

In SEM images, the PDO suture structure showed longitudinal fissures that ran along the entire suture, as well as degradation in the region of the gelatinous spicules with longitudinal fissures along the suture, at concentrations above 12 mg/ml, after 30 days. Suárez-Vega et al. (2019) ^29^, using microphotographs, observed similar aspects after 24 hours of immersion of PDO sutures in HA, which progressed over the 48- and 72-hour evaluation periods, and the authors pointed out that, as the suture releases its pigment, there is an increase in the empty spaces in the central column of the suture, with a disorganization of the peripheral fibrils with wear along the fiber ^29^. These results were similar to another study by Suárez-Veja et al. (2019a) ^31^. In this study, data on variations in suture diameter and weight were not investigated. However, it should be noted that the use of non-crosslinked HA associated with PDO sutures is not supported by solid scientific evidence and standardized methodology and should be carefully considered in practice to avoid negative impacts.

The findings presented in this investigation contribute to a more accurate understanding of the interaction between HA and PDO, reinforcing that the combination of the two biomaterials should be carried out with careful planning. Simultaneous clinical application can be beneficial when performed in separate planes, as demonstrated in recent studies ^30^ ^,^ ^33^. However, direct contact between high-concentration HA and PDO may reduce the longevity of the suture and negatively impact the lifting effect. This study has limitations, including the small number of fragments per group, the absence of a neutral solution control, and the impossibility of direct extrapolation to the clinical setting. In vivo studies and randomized clinical trials are needed to consolidate protocols that combine HA and PDO safely and effectively.

## Data Availability

The research data are available upon request.
